# Congenital Central Hypoventilation Syndrome in Israel—Novel Findings from a New National Center

**DOI:** 10.3390/jcm12123971

**Published:** 2023-06-11

**Authors:** Yakov Sivan, Yael Bezalel, Avital Adato, Navit Levy, Ori Efrati

**Affiliations:** 1 National CCHS Center & Department of Pediatric Pulmonology, Edmond and Lily Safra Children’s Hospital, Sheba Medical Center, Tel Aviv 5262000, Israel; 2 Adelson School of Medicine, Ariel University, Ariel 4070000, Israel; 3 Faculty of Medicine, Tel Aviv University, Tel Aviv 6997801, Israel; 4 Yad LaNeshima, The Israeli CCHS Patients’ Foundation, Tel Aviv 6927728, Israel; 5 Department of Natural and Life Sciences, The Open University of Israel, Ra’anana 4353701, Israel

**Keywords:** congenital central hypoventilation syndrome (CCHS), polyalanine repeat mutations (PARM), non-polyalanine repeat mutations (NPARM), asystole, radiofrequency cardio-neuromodulation

## Abstract

Background. Congenital central hypoventilation syndrome (CCHS) is a rare autosomal-dominant disorder of the autonomic nervous system that results from mutations in the *PHOX2B* gene. A national CCHS center was founded in Israel in 2018. Unique new findings were observed. Methods. All 27 CCHS patients in Israel were contacted and followed. Novel findings were observed. Results. The prevalence of new CCHS cases was almost twice higher compared to other countries. The most common mutations in our cohort were polyalanine repeat mutations (PARM) 20/25, 20/26, 20/27 (combined = 85% of cases). Two patients showed unique recessive inheritance while their heterozygotes family members were asymptomatic. A right-sided cardio-neuromodulation was performed on an eight-year-old boy for recurrent asystoles by ablating the parasympathetic ganglionated plexi using radiofrequency (RF) energy. Over 36 months’ follow-up with an implantable loop-recorder, no bradycardias/pauses events were observed. A cardiac pacemaker was avoided. Conclusions. A significant benefit and new information arise from a nationwide expert CCHS center for both clinical and basic purposes. The incidence of CCHS in some populations may be increased. Asymptomatic NPARM mutations may be much more common in the general population, leading to an autosomal recessive presentation of CCHS. RF cardio-neuromodulation offers a novel approach to children avoiding the need for permanent pacemaker implantation.

## 1. Introduction

Congenital central hypoventilation syndrome (CCHS) is a rare disorder of the autonomic nervous system (ANS) that results from mutations in the paired-like homeobox 2b (*PHOX2B*) gene located on chromosome 4p12. *PHOX2B* encodes a transcription factor that is important for the development of the ANS. CCHS characteristically manifests after birth with hypoventilation and respiratory insufficiency with hypoxemia and hypercarbia due to chemoreceptor insensitivity [1,2,3]. Additionally, features may include Hirschsprung’s disease (HSCR), cardiac sinus pauses, ocular involvement, temperature sensation and control and increased risk for neural crest tumors. It is estimated that more than 3000 cases have been diagnosed worldwide since 1970, with increased prevalence since 2003 when *PHOX2B* genetic testing became available [4,5,6]; however, this is likely an underestimate [2,4]. Data on disease prevalence have been reported from only a few populations, suggesting an incidence of 1:148,000–1:200,000 live births [7,8].

CCHS is inherited in an autosomal dominant pattern [1,2,6], where about 90% of *PHOX2B* mutations are represented by expansions of a polyalanine tract, encoded by exon 3, of the normal (wild-type) allele from 20 repeats to 24–33 repeats on the mutated alleles. This is collectively defined as polyalanine repeat mutations (PARM) [2,3]. About 10% of CCHS cases result from frameshift, missense and nonsense mutations in one *PHOX2B* allele, while the polyalanine expansion is normal (20 on both alleles), and are defined as non-PARM (NPARM) [2]. PHOX2B is a transcription factor encoded by a three-exons gene, which is a member of the paired family of homeobox proteins localized to the nucleus. It is involved in neural crest cell migration and plays a critical role in early embryonic neuronal formation and differentiation, especially in the ANS from the neural crest cell derivative, and in the development of several major noradrenergic neuron populations and the determination of the neurotransmitter phenotype. Normal function of the *PHOX2B* gene is also required for central nervous chemo-sensitivity (mainly to hypercarbia) postnatally throughout the life span. The physiologic ventilatory control abnormality in CCHS appears to be in the integration of chemoreceptor input to central ventilatory controllers, rather than abnormalities in the chemoreceptors themselves [9].

Most CCHS cases occur de novo [2,3]; hence, parents have normal phenotype and genotype. Cases where one of the parents is a mosaic also occur. The PARMs show a genotype–phenotype relationship [2,9,10]. In CCHS, the central drive is decreased, more during sleep than during wakefulness. Hence, all CCHS patients exhibit hypoventilation and decreased response to hypercarbia during sleep. While milder cases with 24–26 PARM require assisted ventilation only while asleep and during intercurrent infections or after general anesthesia, patients with 27 PARM and higher usually require ventilator support 24 h/day. However, unlike PARM, due to the rarity of CCHS, the fact that NPARMs comprise only about 10% of all CCHS cases, and since each specific NPARM results from a different point mutation, not enough data have accumulated to correlate specific NPARMs with clinical presentation. Recently, an international collaboration looking for genotype–phenotype relationships from 302 NPARM cases showed immense genotypic and phenotypic variability associated with CCHS *PHOX2B* NPARM cases [11]. Patients with *PHOX2B* NPARMs can have highly variable phenotypes, ranging from severe respiratory conditions to mild and even subclinical manifestations that may become evident with an additional internal or external stressor [12,13].

Due to the rarity of CCHS, patients with multi-organ involvement are followed and treated by professionals of various specialties, and different countries and regions implement different strategies. Fortunately, specialized CCHS centers and clinics have evolved, contributing significantly to knowledge and experience that improved and optimized CCHS patient management. Moreover, the clinical and research experience accumulated in these specialized centers has been shared and circulated around the world, allowing small countries and communities to follow and implement large centers’ experience with their designated protocols. This is of main virtue also since CCHS is a multi-organ systems disease that may require the involvement of cardiologists, neurologists, geneticists, neurologists, gastroenterologists, ENT, speech therapists, ophthalmologists, sleep specialists and more. Despite the complexity of the phenotypic manifestations, with good medical multidisciplinary support, CCHS patients reach “normal life milestones”, including attending schools, maintaining employment, marrying and having families [2]. A crucial prerequisite is early diagnosis and devoted management by specialized professionals before hypoxic brain damage occurs.

In Israel, over the years, CCHS patients have been managed mainly for their ventilatory support by pediatric pulmonologists around the country. In January 2018, a national CCHS center was founded in the Edmond and Lily Safra Children’s Hospital, Sheba Medical Center, and organized evaluation, follow-up, treatment, family support and genetic consultation for CCHS patients and their families was initiated, applying US leading centers’ protocols (mainly CAMP in Chicago).

The purpose of this report is to share our experience and present some unique and novel findings that accumulated over a relatively short period of four years.

## 2. Patients and Methods

This study was approved by the local IRB (Helsinki Committee). There was no need for patient consent. We were able to reach and contact all CCHS patients in Israel. This was achieved in several ways:

(a) From the Israeli pediatric pulmonologists (*n* = 60) who follow and manage all CCHS cases. Being a small country, all pediatric pulmonologists join the Israeli Pediatric Pulmonology Society and network and meet five times yearly.
(b) From the database of the Israeli CCHS Foundation (“Yad LaNeshima”, www.cchsisrael.org).
(c) Information on CCHS and the new national center was distributed officially to all four insurance agencies, hospitals and community clinics by a formal statement from the chief manager of the Ministry of Health. Israel employs a free-of-charge national health service and by law, all residents are automatically enrolled in and entitled for medical services.
(d) The information was circulated to all pediatricians (including all sub-specialties and neonatologists) via the Israeli Society of Pediatrics.
(e) Information on CCHS and the new national center was circulated both in a television program and daily newspapers.

All CCHS patients and families were invited for evaluation and treatment in the new national CCHS center. Data collected included demographic information, ethnic origin, genetic results, and the full medical history of patient and family. The patients underwent an initial clinical and laboratory evaluation by specialists of the following disciplines:

Cardiology: cardiac echocardiogram, ECG and 72 h Holter recording; radiology: chest radiography and abdominal ultrasound; ENT: tracheoscopy for those who had a tracheostomy; respiratory: pediatric pulmonologist and pediatric intensivist, gas exchange measurement and ventilator adjustment. Other disciplines were involved according to patients’ clinical presentation. These disciplines are services in the Safra Children’s Hospital, which is a part of the general Sheba Medical Center. Each of these disciplines agreed to consult, follow and treat all patients whenever a problem in their field needed their assistance. All these services have received a detailed explanation and talks on CCHS with specific relation to their field and also increased their knowledge from the literature whenever needed. Additionally, consultation with experienced centers overseas (mainly CAMP) took place when needed. Polysomnography was performed in patients who did not have recent sleep evaluation. Follow-up included 6–12 months of scheduled visits that included repeat assessment as detailed above.

All patients under 18 years old are followed in the community by a dedicated physician experienced in home ventilation who in addition to scheduled home visits is available for phone consultations and home visits as needed.

The staff of the new CCHS center includes one doctor and one coordinator nurse both at 30% of their time (1.5 days/week). The staff of the CCHS center is available to the patients 24/7 for assistance by phone and patients are seen, in addition, whenever a medical problem arises that requires in-house assessment. Being recognized as a national center, all relevant hospital services for CCHS are available to all patients and insurance coverage is guaranteed by law.

## 3. Results

**Figure 1:**
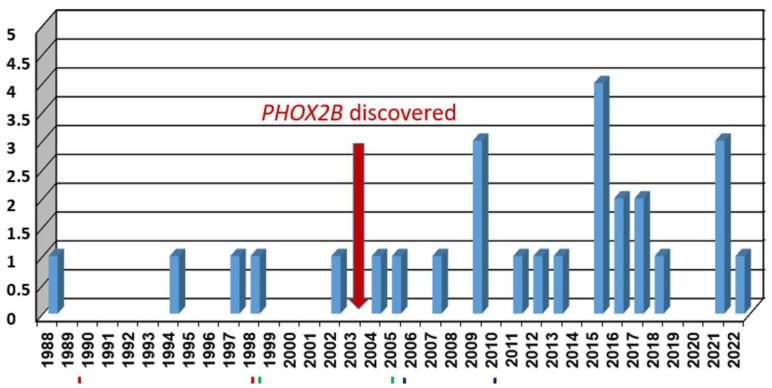
CCHS—new cases by year of birth.

Twenty patients were located at the time of the national CCHS center foundation (January 2018). Age ranged between 1 and 33 years. Over the following four years, seven new patients were diagnosed at birth and joined the center (male: 16 of 27). Patients’ distribution by year of birth is presented in Figure 1.

Several novel and unique findings were observed.

### 3.1. Incidence

The overall rate of new CCHS cases over the last 20 years (since gene discovery) is 1:166,000 live-births with a rate of 1:127,000 for the Jewish population—almost twice the reported rate from other countries [4]. Interestingly, and with adjustment to the natural increase in number of births per year, the rate for the years 2003–2006 was 1:240,000 and increased to 1:165,000 for 2007–2014 and to 1:148,000 for 2015–2022. The rate for the Jewish population was 1:166,000, 1:149,000 and 1:109,000 for the three periods, respectively. Three patients were from Arabic Islamic ethnic origin: one Israeli, one from the Palestinian Authority, one from the city of Gaza. The latter two were not included in the rate calculation. 9 patients out of 27 (33%) were ultra-orthodox Jews, which accounts for 9 of 24 (39%) Jewish ultra-orthodox patients out all Jewish cases, while the average prevalence of that population over the last 20 years is only 11% of the general society and 14% of the Jewish population.

### 3.2. Genotype

**Figure 2:**
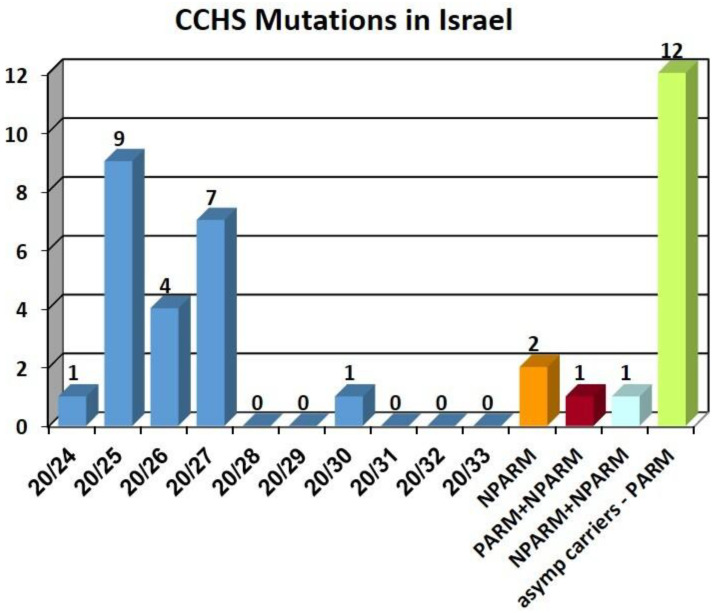
Distribution CCHS mutations.

**Figure 3:**
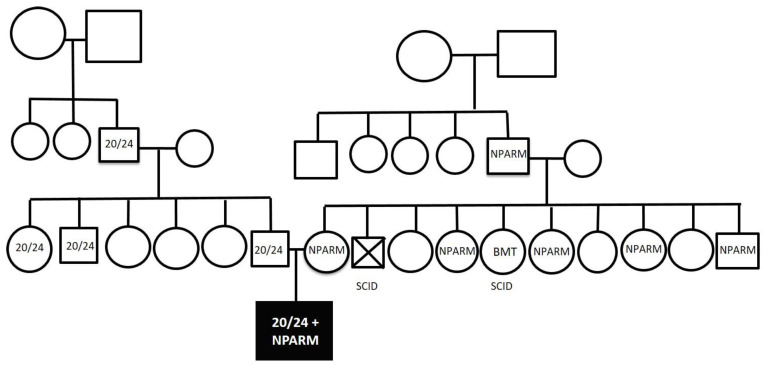
Pedigree of the family. Squares indicate males and circles indicate females. The proband is indicated by the black square. SCID—severe combined immune deficiency. BMT—bone marrow transplantation. The marked square with X indicates an uncle (mother’s brother) who died of SCID. Adapted with permission from reference [15].

Overall, the distribution of mutations was similar to the distribution reported from countries with large populations [14]. A total of 25 patients out of 27 (93%) had an autosomal dominant genotype, where 23 of 27 (85%) patients had a PARM on a single *PHOX2B* allele with the most common mutations being 20/25, 20/26 and 20/27 PARM, and 2 patients (7%) had an NPARM on one allele. An additional two patients (7%) had an unusual novel recessive genotype with a full respiratory phenotype (Figure 2). One of these carries a co-occurrence of 20/24 *PHOX2B* PARM on one allele and a new NPARM missense variant on the other allele (NM_003924.4:c.785G>T, p.Gly262Val). The two parents and 50% of their siblings as well as both grandfathers of both sides carry these PARM or NPARM mutations, respectively, being completely asymptomatic (Figure 3). This case has been reported in detail previously [15]. The other case was homozygote for two identical NPARMs on both *PHOX2B* alleles. This mutation is the same as the missense variants found in the previous recessive genotype patient (NM_003924.4:c.785G>T, p.Gly262Val). Both parents were found to carry this NPARM on one allele only and are completely asymptomatic. Hence, in both cases CCHS follows an autosomal recessive inheritance pattern.

### 3.3. Genotype–Phenotype Relationship

While patients with PARM followed the previously described genotype–phenotype relationship, patients with NPARM and the homozygote patients with the autosomal recessive genotype had unpredictable and unique clinical presentation and course. The patient with the NPARM/24 mutation requires assisted ventilation when asleep, but no other clinical or laboratory manifestations have been observed over seven years. The patient with the recessive homozygote NPARM CCHS had a difficult course since birth, including 24/7 respiratory support and gastrointestinal and cardiac involvement.

The two NPARM patients with a “classical” autosomal dominant (heterozygote) NPARM genotype presented unique clinical manifestations. One was a seven-year-old boy, born in Gaza. His parents are second- degree cousins. At the age of three months, he was transferred to our pediatric oncology department due to chest and abdominal masses, which were diagnosed as neuroblastoma. Further evaluation due to suspected color change verified CCHS due to a novel heterozygous *PHOX2B* NPARM (c.369delC p.Pro123fs) in exon 2 of the *PHOX2B* gene, within the region encoding the homeobox domain of the protein. Initial respiratory evaluation during sleep showed no desaturations below 91% and CO_2_ levels up to 45 mmHg. Due to the lack of facilities and experts in his neighborhood, the parents decided not to consider assisted respiratory support but to monitor nocturnal oxygen saturation. He responded to his hemato-oncologic treatment. However, over the years, respiratory assessment showed gradual worsening with recent evaluation showing desaturations down to 85% and hypercapnia up to 54 mmHg during sleep. Assisted ventilator support during sleep was started. Repeat examinations showed no cardiac, GI or other clinical manifestations.

**Figure 4:**
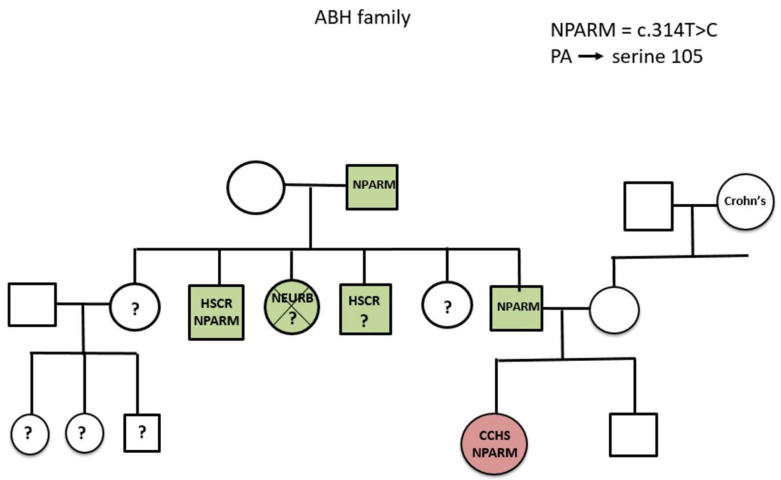
Pedigree of family. Squares indicate males and circles indicate females. HSCR—Hirschsprung’s disease, Neurb—neuroblastoma.

The other NPARM patient was an eight-year-old girl that carried another novel *PHOX2B* mutation, a c.314T>C transition that causes a phenylalanine to serine missense, at position 105 within the homeobox domain of the PHOX2B protein (c.314T>C, F105S). This mutation was also detected in other family members with no disease manifestation (Figure 4). This patient has Hirschsprung’s disease. Repeated polysomnography showed transient hypercapnia up to 50 mmHg with oxygen saturations of mostly 95–97% and no desaturations below 91%. Hence, the parents decided to continue with saturation monitoring during sleep alone.

### 3.4. Cardio-Neuromodulation by Radiofrequency Ablation for Cardiac Asystoles

A nine-year old boy with 20/27 PARM CCHS had recurrent asymptomatic episodes of sinus pause documented in a yearly routine of 72-hour Holter monitor recording. There were overall 23 pauses of 3–4 s during both sleep and awake time. Three repeat Holter recordings showed similar findings. The patient is appropriately ventilated during sleep through a tracheostomy since birth. Of note, the parents reported two recent seizure episodes.

A permanent pacemaker (PPM) implantation was considered. However, given the relatively high rate of complications and the effects on quality of life associated with cardiac pacing, we applied a novel approach based on the idea that the sinus pauses resulted from increased vagal tone. A cardio-neuromodulation of the sinus node using radio-frequency (RF) ablation of the anterior right-ganglionated plexi was performed using a technique that has previously been reported only in adults [16]. The procedure was performed using a retrograde-arterial and a right-atrial-venous approach, guided by the merged image of an electro-anatomical mapping (CARTO^®^ mapping system; BIOSENSEWEBSTER) and of a pre-procedural cardiac computed tomography. Baseline intravenous injection of 1 mg (0.04 mg/kg) atropine resulted in a 40 beats per minute (bpm) increase in heart rate. RF ablations of the high postero-septal right atrial (RA) region opposite to the right superior pulmonary vein and the high anterior septum part opposite the aorta were performed. Post-ablation intravenous atropine had no effect on heart rate, consistent with a successful endpoint. In addition, following the ablation procedure an implantable loop recorder (ILR) was inserted and programmed to record any R-R interval longer than 3 s.

Over 36 months of follow-up with an ILR and repeated Holter monitor recordings, the patient has been asymptomatic with no documented bradycardic episodes. His documented average heart rate is 104 bpm and the minimum heart rate was 73 bpm. Of note, no further seizure episodes occurred, suggesting that these events were indeed syncope episodes. A cardiac pacemaker was avoided.

## 4. Discussion

Our data provide several new findings that may add to clinical practice and basic understandings in CCHS. We nevertheless believe that the main message of our experience is that a significant additional benefit arises from a nationwide or regional expert-based CCHS center that covers the entire country or region, depending on the population size, for both clinical and basic purposes. Our findings show that data from even a small country with a small cohort may provide new important information on CCHS over a relatively short period. This may be of major importance due to the rarity of the disease and the benefit of a designated professional center. The recognition that rare diseases, and specifically CCHS, need to be consolidated to maximally learn from the children and to optimally improve the care of the affected children, and that the ability to study cohorts of children with CCHS at these centers leads to the major advances that follow has been highlighted by Weese-Mayer et al. in 2009 [14]. Our findings strongly support this conclusion. Additionally, our experience shows that a center with minimal manpower may cope with a population of 8–9 million people when all the services of the various relevant disciplines are available, cooperative and covered by law.

While only five patients were diagnosed over the 15 years 1988–2003 (0.33 cases/year), 27 patients were diagnosed over the 18 years 2004–2022. This is obviously the result of increased awareness and especially the discovery of the *PHOX2B* gene and the capability for laboratory diagnosis that started in 2003 [5,6].

The distribution of the PARMs and the incidence of NPARM in our population is similar to the distribution described from 640 cases from several populations and countries [14], suggesting that this is indeed the spread of CCHS worldwide. More information from other countries is, therefore, very valuable.

We faced a significant practical problem to persuade some of our patients and family members to cooperate and follow the recommended clinical practices and to agree to evaluate their asymptomatic family members. This was apparent in two groups: ultra-orthodox Jewish families, and older patients. Interestingly, the occurrence of ultra-orthodox Jewish patients in our cohort is much higher than their occurrence in the general population (33% of the cohort and 39% of Jewish CCHS cases compared to 11% and 14%, respectively). We have no explanation for this observation and CCHS has not been found to be more frequent in ultra-orthodox Jewish people from other countries with a large Jewish population.

Some ultra-orthodox Jewish patients and their family members only partially followed our clinical recommendations and were not interested in genetic consultation, stating that they will never stop or hold pregnancy anyway, and hence amniocentesis in following pregnancies was unacceptable. This was due to religious beliefs and the instructions of their rabbis. Fortunately, some of these families agreed to preconception diagnosis. Nevertheless, some ultra-orthodox families were reluctant to perform any family screening for *PHOX2B* tests. This emphasizes the need to adjust practices to specific populations and receive help from religious authorities, such as Jewish rabbis whom ultra-orthodox Jewish follow and obey much more than their cooperation with medical experts. Indeed, collaborating with some rabbis was partially successful in convincing family members to agree to *PHOX2B* testing, albeit limited to the parents and only some of the patients’ and parents’ siblings.

The other challenging group was older patients who had not received designated care, explanation and comprehensive clinical support before the start of our program. For example, it was difficult to convince patients 20 years old and older to start periodic 72-h Holter recordings after this had not been performed before; consequently, some did not follow the recommendations. This highlights the need to establish authorized and recognized national or regional CCHS centers early, in addition to investing in increasing the awareness of the relevant colleagues nationwide.

Some new interesting understandings may be derived from the two cases with a recessive CCHS genotype, whose multiple heterozygous family members do not manifest any CCHS symptoms. Hence, the c.785G>T variant on *PHOX2B* exon 3 results in a glycine to valine substitution at position 262, which is the second amino acid right after the polyalanine repeat. Although glycine is a neutral and non-polar amino acid and valine has a hydrophobic side chain, in-silico analyses suggest that this variant is likely to be tolerated. However, segregation of this variant among members of two Israeli families indicates that one allele of this variant may be insufficient to cause clinically apparent disease, thus presenting a true autosomal recessive pattern of CCHS, which is unusual for this syndrome, although it has been described for the 20/24 PARM when present in homozygosity [17]. It is speculated that the specific c.785G>T NPARM causes subthreshold decrements in *PHOX2B* functioning and when present on both alleles there is sufficient impairment of the PHOX2B protein function to cause clinical expression of the CCHS phenotype.

This c.785G>T variant is present in population databases (rs768420488, ExAC 0.003%). The finding of the same mutation in the two patients reported here and the observation of several asymptomatic carriers in both families suggests that this NPARM may be much more common in the population and probably in a specific population. This homozygote NPARM was not observed in a recent large study of 302 CCHS cases with NPARM [11]. At present, the rarity of the cases does not seem to justify preconception screening; nevertheless, this position may change if additional unrelated cases are reported, especially in a specific population, since asymptomatic heterozygotes have been found. Additionally, the fact that previous cases carrying this NPARM have not been reported suggests that unlike most NPARMs this specific mutation in one allele does not cause clinical disease unless the other *PHOX2B* allele also harbors a PARM or an NPARM.

The fact that heterozygote family members with the novel missense NPARM are completely asymptomatic while when this NPARM combines with a 24-polyalanine expansion or with the same NPARM on the other allele, it presents as a CCHS phenotype, may suggest that with this specific NPARM the amount of undamaged protein from one allele is enough for normal life, and that this specific NPARM does not have a toxic gain of function. This presents a true autosomal recessive pattern. Obviously, we do not know the prevalence of this NPARM in the general population. The finding of asymptomatic family members with 20/24 PARM is not surprising, since this PARM has been reported to present with either clinically CCHS from birth, late-onset CCHS or as asymptomatic carriers [2,15,18,19,20]. It is speculated that the 20/24 PARM may also cause a non-clinically important decrease in the PHOX2B protein that decreases further to impair function when the other allele is also mutated.

Although most CCHS cases occur de novo, phenotypic variability when the mutation was inherited from an asymptomatic or a mild phenotypic parent has been reported especially with NPARMs and the 20/24 PARM [3,15,21,22].

The two heterozygote (autosomal dominant) NPARM cases are also unique in that both do not receive assisted ventilation. These patients were introduced to us when they already were three and four years old and the parents were reluctant to consider assisted ventilator support with or without tracheostomy that will significantly affect their quality of life. Interestingly, although CCHS patients due to NPARM are usually considered to have a worse clinical course, and most require assisted ventilator support also when awake, at present, these two patients grow and develop well without assisted ventilation. However, the patient from Gaza, who at the beginning showed reasonable gas exchange including during sleep, progressed to hypoventilation over time and assisted ventilation during sleep has been recently applied.

The other heterozygote NPARM case showed variable penetrance in several family members carrying the same NPARM. Variable penetrance was previously reported in a family with three generations carrying a novel NPARM with both severely affected and asymptomatic members having the NPARM [21]. This variable penetrance in our and the abovementioned families supports the hypothesis that the CCHS phenotypic manifestations may be influenced by environmental or genetic modifiers that presently are unknown.

Our approach to performing a right-sided cardio-neuromodulation by ablating the parasympathetic ganglionated plexi area using radiofrequency energy in a CCHS patient with sinus pauses is novel. Prolonged sinus pauses were documented in this population and are associated with an increased risk of sudden cardiac death, where 83% of the carriers of the *PHOX2B* 20/27 PARM mutation will suffer from sinus pauses longer than 3 s at some point. Hence, in order to detect sinus pauses before they present clinically, an annual 72-hour Holter recording is recommended [23]. Given their increased risk of sudden death, the common practice in this population is to insert a cardiac pacemaker when prolonged pauses (>3 s) are observed, even when symptoms have not yet occurred [4,23].

Radiofrequency (RF) ablation of right-sided parasympathetic ganglionated plexi (PGPs) is a novel approach to treat neurally-mediated syncope and functional sinus node dysfunction [16]. This method has been implemented merely in the adult population. Targeting the anatomically known locations of the PGPs affecting the sinus and AV nodes causes a vagolytic effect, with a subsequent increase in sinus rhythm and enhancement of AV conduction, respectively.

Only two pediatric cases of RF ablation for vagal denervation were reported thus far from Brazil and the US [24,25]. Both were 15–16-year-old adolescents with recurrent syncope episodes. There were no previous reports of such a therapeutic approach in a CCHS patient. The latter population displays a variety of associated cardiovascular symptoms, namely attenuated heart rate variability, increased incidence of atrial and ventricular ectopy, sinus bradycardia and prolonged sinus pauses [22,26,27]. Since the chances of future sinus pauses in CCHS cases increases with age, their only therapeutic option until now was a permanent pacemaker implantation. The observation that continuous monitoring for 36 months following the procedure did not document bradycardic events (no R-R interval longer than two seconds) is reassuring and supports that the mechanism behind the episodes is vagally-mediated rather than intrinsic degenerative disease of the conduction system.

Avoiding permanent pacemaker implantation in children and young adults with CCHS is of paramount importance, since these patients also suffer from many other major problems. In addition to avoiding the insertion procedure, RF ablation for vagal denervation may prevent lifelong pace-maker complications such as infection, reverse ventricular remodeling, ventricular dysfunction and the need for hardware exchange. Obviously, there is a need for additional experience and controlled studies to assess this therapeutic option, especially in CCHS patients. Interestingly, this patient did not have any further seizure episode. We speculate that the two seizure events he had prior to the procedure might have been syncope-related rather than of primary neurologic origin.

## 5. Summary and Conclusions

A significant additional benefit arises from a nationwide or regional expert CCHS center both for clinical and basic purposes. Data from a small center with limited resources may provide new important information on CCHS. The rate of CCHS in some populations may be higher than reported. Asymptomatic NPARM mutations may be much more common in the general population than previously known, leading to an autosomal recessive presentation of CCHS. Whether such mutations, and specifically the c.785G>T, p.Gly262Val NPARM, should be screened in population where it has been found waits for additional findings. RF cardio-neuromodulation offers a novel approach to children and may be specifically beneficial to CCHS patients with cardiac pauses, avoiding the need for permanent pacemaker implantation.

## Data Availability

Not applicable.
